# Cellulose Nanofibrils vs Nanocrystals: Rheology of Suspensions and Hydrogels

**DOI:** 10.3390/gels11110926

**Published:** 2025-11-19

**Authors:** Alexander S. Ospennikov, Alexander L. Kwiatkowski, Olga E. Philippova

**Affiliations:** Physics Department, Lomonosov Moscow State University, 119991 Moscow, Russia; ospennikov@polly.phys.msu.ru (A.S.O.); kvyatkovskij@physics.msu.ru (A.L.K.)

**Keywords:** cellulose nanocrystals, cellulose nanofibrils, rheological properties, hydrogels, crosslinking

## Abstract

Plant-derived nanocellulose particles, such as cellulose nanofibrils (CNFs) and cellulose nanocrystals (CNCs), are becoming increasingly popular for a wide range of applications. In particular, when they are employed as rheology modifiers and/or fillers, a choice between CNFs and CNCs is often not obvious. Here, we present the results of a comparative study on the rheological properties of suspensions and gels of carboxymethylated CNFs and CNCs with the same surface chemistry, surface density of charged groups, and thickness. We demonstrate that, at the same weight concentration, CNF suspensions have much higher viscosity and storage modulus, which is due to their longer length providing many entanglements. However, when comparing at the same nanoparticle concentration relative to C*, the situation is reversed: viscosity and storage modulus of CNCs appear to be much higher. This may be due in particular to the higher rigidity and intrinsic strength of highly crystalline CNCs. The gel points for CNF and CNC suspensions (without crosslinker) were compared for the first time. It was found that in the case of CNFs, the gel point occurs at a 3.5-fold lower concentration compared to that of CNCs. Hydrogels were also obtained by crosslinking negatively charged nanocellulose particles of both types by divalent calcium cations. For the first time, the thermodynamic parameters of the crosslinking of carboxymethylated CNFs by calcium ions were determined. Isothermal titration calorimetry data revealed that, for both CNFs and CNCs, crosslinking is endothermic and driven by increasing entropy, which is most likely due to the release of water molecules surrounding the interacting nanoparticles and Ca^2+^ ions. The addition of CaCl_2_ to suspensions of nanocellulose particles leads to an increase in the storage modulus; the increase being much more significant for CNCs. Physically crosslinked hydrogels of both CNFs and CNCs can be reversibly destroyed by increasing the shear rate and then quickly recover up to 85% of their original viscosity when the shear rate decreases. The recovery time for CFC networks is only 6 s, which is much shorter than that of CNC networks. This property is promising for various applications, where nanocellulose suspensions are subjected to high shear forces (e.g., mixing, stirring, extrusion, injection, coating) and then need to regain their original properties when at rest.

## 1. Introduction

Due to increasing environmental concerns regarding synthetic polymer waste, there is a growing need for new, eco-friendly biodegradable materials from natural resources. In this regard, cellulose-based nanomaterials have the potential to become one of the most promising sustainable materials due to their unique properties, renewable nature, and wide availability [1]. The global market of cellulose-based nanomaterials is expected to grow from USD 1.07 billion in 2025 to USD 6.16 billion by 2034 [2].

Cellulose-based nanomaterials, also known as nanocellulose, include, in particular, cellulose nanofibrils (CNFs) and cellulose nanocrystals (CNCs) [3]. CNFs are the smallest fibrous component of wood, measuring around 4–20 nm in width and up to a few micrometers in length [4,5]. They consist of alternating amorphous and crystalline domains, although the amorphous regions can be quite small [6]. CNFs are usually produced from wood and plants using mechanical disintegration after an appropriate pretreatment, such as carboxymethylation, carboxylation, or sulfonation, among others [7]. The appearance of charged groups during pretreatment reduces the attraction between fibrils, facilitating their disintegration and further stabilizing suspensions of CNFs.

In their turn, CNCs represent rodlike nanocrystals that are approximately 4–70 nm wide and 100–500 nm long [8]. Within CNCs, macromolecules are arranged in a parallel pattern with a helical twist and are held together by hydrogen bonds [9]. Usually, CNCs are produced through acid hydrolysis of cellulose materials, which selectively breaks down the amorphous domains, leaving behind rodlike nanocrystals [10]. During this process, charged groups appear on the surfaces of CNCs, which stabilize suspensions of nanocrystals in water through electrostatic repulsion between similar charged CNCs. CNCs have very high crystallinity of up to 88% [11] and therefore can offer enhanced mechanical properties. For instance, their elastic modulus is estimated at 110–220 GPa and tensile strength at 7.5–7.7 GPa [12].

CNFs and CNCs are often used as nanofillers for reinforcing various polymeric matrices [13,14,15,16], but they can also be used to form materials on their own, such as gels.

CNFs are very long and can entangle with each other, forming hydrogels even at low concentrations (<1 wt%) [7]. These hydrogels are stabilized by hydrogen bonding and van der Waals interactions without any crosslinkers [17,18], but they are rather weak. Hydrothermal treatment (heating in a sealed reactor) can be used to reinforce such gels [19]. Stronger gels can be obtained by crosslinking negatively charged CNFs by multivalent cations [20,21]. These cations can induce an increase in storage modulus G′ of carboxylated CNFs by up to 4 orders of magnitude [20]. Density functional theory (DFT) calculations have shown that the crosslinking of carboxylated cellulose fibrils by Ca^2+^ ions is due to electrostatic interactions [22]. The involvement of CNF carboxylate groups in the interaction with cations has been demonstrated by a shift in the infrared absorption band corresponding to the symmetric stretching vibrations of the COO^−^ group when the cations are added [20].

As for CNC gels, they can also be prepared without additives, but it requires either a high concentration of nanocrystals [23] or a sophisticated treatment, such as freeze–thaw cycling with [8] or without hydrothermal treatment [24]. Using multivalent ions as crosslinkers allows one to prepare stiff CNC gels at moderate concentrations (3–4 wt%) [25,26]. Visualization of the gel structure by cryo-electron microscopy (cryo-EM) and cryo-electron tomography has revealed that multivalent cations enhance the aggregation of CNCs, leading to the growth of aggregates in both length and thickness [26]. This stiffens the network of CNCs. It was observed that the strength of CNC hydrogels increases with the charge number of the crosslinker (Al^3+^ > Mg^2+^ > Na^+^) and with the ionic radius of the crosslinkers bearing the same charge (Sr^2+^ > Mg^2+^ > Ca^2+^) [25]. However, for CNFs mixed with poly(vinyl alcohol), the order of multivalent cations that lead to stronger gels is reversed: Ca^2+^ > Cu^2+^ > Zn^2+^ > Al^3+^ > Fe^3+^ [27]. In this case, Ca^2+^ provides the strongest gel.

Among different multivalent cations used for nanocellulose crosslinking, Ca^2+^ ions seem to be one of the most promising. These ions are non-toxic and biocompatible [28], which opens the possibility of using the resulting hydrogels in medical applications. At the same time, they provide strong, but still reversible crosslinks [26,29,30].

The reversibility of gelation is an important property. Indeed, suspensions and gels of cellulose nanoparticles usually undergo different processes such as mixing, stirring, extrusion, pumping, injection, and coating, where they are exposed to high shear rates and should recover their initial viscosity at rest. Moreover, the reversibility of gelation is exploited in such applications as 3D extrusion printing [17] or the use of injectable hydrogels in medicine [18]. The reversibility of CNCs/Ca^2+^ gels was demonstrated recently [26]. It was shown that they recover up to 90% of their viscosity in 15 s when the shear rate is decreased. This requires a high concentration of the crosslinker (two Ca^2+^ per one COO^−^ group) [26]. The reversibility of CNFs/Ca^2+^ gels was also demonstrated [29,30]. However, the viscosity recovery was not quantitatively characterized—neither the exact recovery time nor the percentage of recovery were reported, which does not allow for a comparison between the recovery characteristics of CNF and CNC gels.

At the same time, for a proper selection of components for nanocellulose-based gels for different applications, it is important to compare the rheological properties of CNC and CNF suspensions and gels, including the reversibility of gelation—that is, the time and completeness of viscosity recovery.

Although many papers, including reviews, describe the properties of CNFs and CNCs [5,31,32,33], there are only a few comparative studies of the rheological properties of CNF and CNC suspensions with similar characteristics (e.g., similar origin, surface chemistry, etc.). Li et al. [34] compared CNFs and CNCs derived from the same cellulose material: CNCs were obtained from CNFs by sulfuric acid hydrolysis. As a result, although being of similar origin, the cellulose nanoparticles had different surface chemistries: carboxylate groups appeared on CNFs due to delignification, and sulfogroups appeared on sulfuric acid-hydrolyzed CNCs. The charge content was also different, with zeta potential values of −4.6 mV for CNFs and −12.8 to −35.4 mV for CNCs. Based on the experiments, the authors proposed different structures for CNF and CNC networks formed at high nanoparticle concentrations. They suggested that the network in CNF suspensions is mainly due to physical entanglements between CNFs. In contrast, the network of sulfuric acid-hydrolyzed CNC is formed by interactions between nanocrystals, as short and rigid CNCs have less potential to form a physically entangled network.

Thus, the present paper is aimed at the study of the rheological properties of suspensions and hydrogels of carboxymethylated CNFs and CNCs that have similar surface chemistry, the same content of charged groups, and the same thickness. Hydrogels were formed either without additives or by crosslinking negatively charged cellulose particles by non-toxic and biocompatible divalent calcium ions. The rheological properties of CNFs and CNCs were compared both before and after gelation. Particular attention was paid to the reversibility of Ca^2+^-induced gel formation and the thermodynamics of Ca^2+^/COO^−^ interactions, which are responsible for crosslinking.

## 2. Results and Discussion

### 2.1. Characteristics of Nanoparticles

The CNFs under study have the length L of 1–3 microns and an average diameter D of about 4 nm, which corresponds to a very high aspect ratio L/D of ca. 500. The CNCs used for comparison have a similar diameter (D = 5 nm), but a shorter length (L = 90 nm), resulting in a lower aspect ratio L/D of ca. 20.

**Figure 1:**
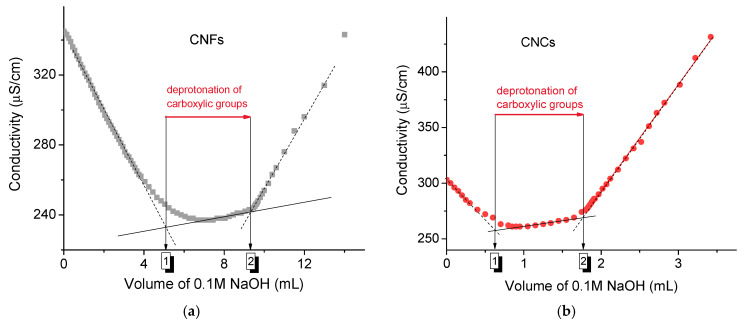
Conductometric titration curves of aqueous suspensions of CNFs (0.3 g) (**a**) and CNCs (0.1 g) (**b**) with 0.1 M NaOH. 1—strong acid equivalence point, 2—weak acid equivalence point. The volume of NaOH added between the strong and weak acid equivalence points, which was consumed for the deprotonation of carboxylic groups, was used to estimate the content of these groups.

The amount of charged COO^−^ groups on the surface of cellulose nanoparticles was estimated by conductometric titration after adding excess HCl to protonate all the carboxyl groups. The titration curves consisted of three parts (Figure 1). An initial decrease in conductivity is due to the consumption of protons from the strong acid HCl by NaOH. This is followed by a plateau, which is attributed to the deprotonation of carboxylic groups [35]. The further increase in conductivity is associated with the increase in OH^−^ ions as NaOH is added in excess. The difference in the volume of NaOH added between the strong and weak acid equivalence points was used to estimate the content of carboxylic groups, which was 1.25 ± 0.10 mmol per gram for CNFs (Figure 1a) and 1.15 ± 0.10 mmol per gram for CNCs (Figure 1b).

**Figure 2:**
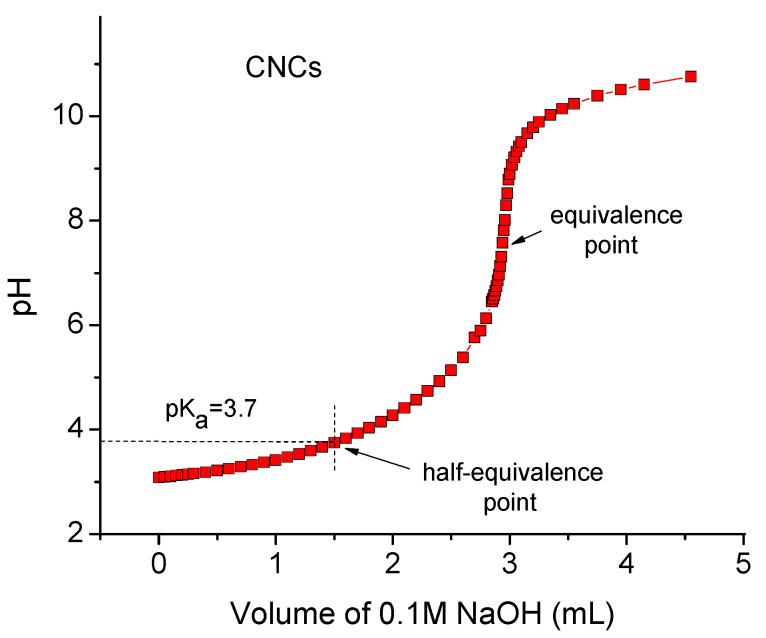
pH titration curve of 0.05 wt% aqueous suspension containing 0.1 g CNCs with 0.1 M NaOH. The pK_a_ value is determined as pH at half-equivalence point.

From the potentiometric titration curve (Figure 2), the pK_a_ of the surface carboxylic groups in the CNCs was determined as the pH value at the half-equivalence point, when half of the COO^−^ groups have been protonated [36]. The pK_a_ for carboxylic groups in the CNCs was found to be approximately 3.7 (Figure 2), which is close to the pK_a_ value (pK_a_ = 3.9) reported for carboxylated CNFs [37]. Thus, at pH 6.5, which was used in our experiments, all carboxylic groups in CNF and CNC suspensions were deprotonated, and both types of nanoparticles had almost the same content of charged groups per gram.

### 2.2. Before Crosslinking

#### 2.2.1. Phase Behavior

The CNF and CNC aqueous suspensions exhibit high stability. They do not undergo phase separation over the entire range of concentrations studied (up to 4 wt% CNFs and 8 wt% CNCs). This may be due to their high surface charge. The high charge provides colloidal stability to the suspensions due to the increased electrostatic repulsion between particles [38]. A good colloidal stability of CNFs may also be due to a steric hindrance effect, caused by the kinks in the fibrils preventing neighboring fibrils from coming into close contact and forming bundles [39].

#### 2.2.2. Viscosity

**Figure 3:**
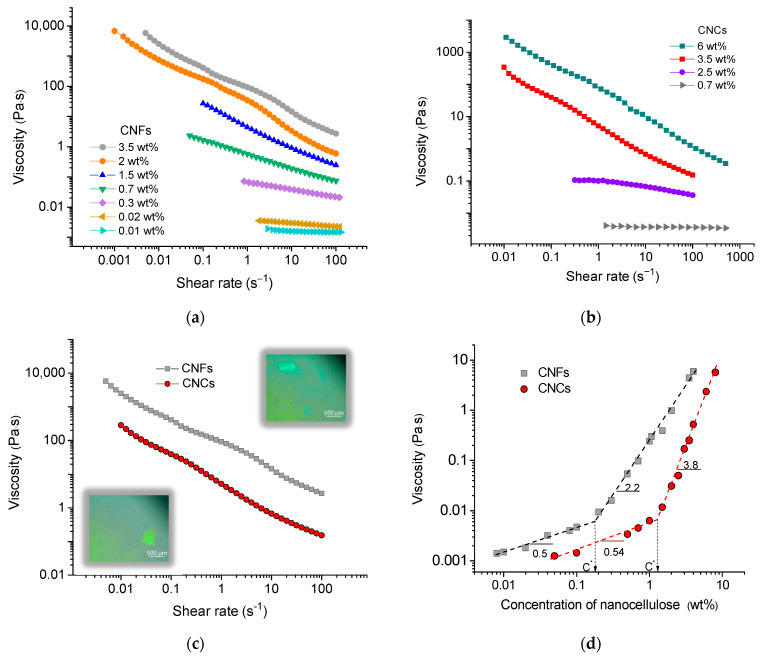
(**a**) Flow curves for aqueous suspensions of CNFs of different concentrations indicated in Figure. (**b**) Flow curves for aqueous suspensions of CNCs of different concentrations indicated in Figure. (**c**) Flow curves for 3.5 wt% aqueous suspensions of CNFs (squares) and CNCs (circles). Inset: Polarized optical microscopy images of same CNFs (top) and CNCs (bottom) suspensions at rest (without flow). (**d**) Dependencies of viscosity on CNF and CNC concentration at shear rate of 45 s^−1^. Arrows indicate overlap concentrations C* corresponding to onset of percolation of nanoparticles.

Figure 3a shows the flow curves for CNF suspensions with different concentrations ranging from 0.01 to 4 wt%. One can see that at low concentrations (0.01–0.02 wt%), the suspensions exhibit almost Newtonian behavior, meaning that their viscosity does not change with the shear rate. More concentrated samples (up to 1 wt%) demonstrate a shear-thinning behavior, which can be related to the alignment of fibrils along the direction of shear. A peculiar type of flow curve is observed at CNF concentrations exceeding 2 wt%. Under these conditions, two shear thinning regions with different slopes can be identified (Figure 3a). They are similar to those previously reported for some CNF suspensions [40,41].

Three similar types of flow curves are also observed for the CNC suspensions (Figure 3b): Newtonian curves at low concentrations (below 1 wt%), one-slope shear thinning curves at intermediate concentrations, and two-slope shear thinning curves at concentrations higher than 3 wt%. They are in accordance with data from the literature [26,42,43]. So, both kinds of cellulose nanoparticles demonstrate a peculiar type of flow curve with two shear-thinning regions when the concentration of nanoparticles exceeds a certain threshold.

The two-slope flow curves are often observed in lyotropic polymer liquid crystals (LCs) [44]. The first shear thinning region (at low shear rates) is assigned to the alignment of LC domains along the direction of flow, while the second shear thinning region (at high shear rates) is attributed to the alignment of individual particles [42]. Such behavior was demonstrated for CNC suspensions by small-angle light scattering under shear flow in combination with rheo-small-angle X-ray scattering [45]. For CNC suspensions, the appearance of two-slope flow curves correlates well with that of birefringence [26,43]. As for CNF suspensions, in the literature, sudden slope changes in CNF suspensions are often associated with the formation and breakdown of shear-induced structures [7]. In the present system, the onset of birefringence is detected at slightly lower concentration (1.4 wt%, Figure S1) compared to the appearance of the two-slope flow curve in rheology (2 wt%). Therefore, the origin of the two slopes in CNF suspensions may not be exactly the same as in CNC suspensions.

Figure 3c compares the flow curves for CNF and CNC suspensions at the same concentration (3.5 wt%). At this concentration, the LCs are observed in both systems at rest, as evidenced by the polarized optical microscopy images at crossed polarizers provided in the insets. One can see that in the CNF suspension, the change in slope is observed at a much higher shear rate, suggesting that the disruption of LC domains in this system requires higher shear. Note that a similar tendency (increase in the shear rate corresponding to the change in slope of the flow curve) is observed at increasing concentration of CNFs (Figure 3a). As at higher concentrations of CNFs, there are more entanglements, a higher shear rate is needed to destroy the aggregates responsible for the first slope.

Figure 3d shows a comparison of the dependencies of viscosity η on nanoparticle concentration for CNF and CNC suspensions. Viscosity is taken at a shear rate of 45 s^−1^, which corresponds to the second slope of the flow curves, where the flow is dominated by individual particles. Both dependencies have two distinct concentration regions corresponding to dilute and semidilute suspensions (Figure 3d). The transition between these two regions occurs at the overlap concentration C*. One can see that the C* value for the CNF suspension (0.175 wt%) is much lower than that for the CNC suspension (1.3 wt%). This is expected, given their longer length L, since C*~L^−2^ [4].

Above C*, the CNC suspension demonstrates a much stronger increase in viscosity compared to CNFs: η ~ C^3.8^ vs. η ~ C^2.2^. These power law dependencies are consistent with those reported previously for carboxymethylated CNCs [26] and CNFs [29,41,46] suspensions in the semidilute regime. The stronger increase in viscosity for CNCs may be due to their high rigidity. For CNCs, the concentration dependence of viscosity is expected to follow the theoretical prediction for semidilute solutions of rods: η ~ C^3^ [47]. The somewhat higher exponent observed for CNCs may be due to the rod jamming effect [48] and the growth of CNC fibrillar-like aggregates occurring at increasing concentration of nanocrystals, which was demonstrated by cryo-EM and cryo-electron tomography previously [26]. While the viscosity scaling for CNCs can be explained by a theory for rotational Brownian motion of rigid rods as a whole, the CNFs have flexibility, which should contribute to their dynamics [49], lowering the exponent in the η ~ C^n^ scaling relationship.

#### 2.2.3. Dynamic Mechanical Properties

**Figure 4:**
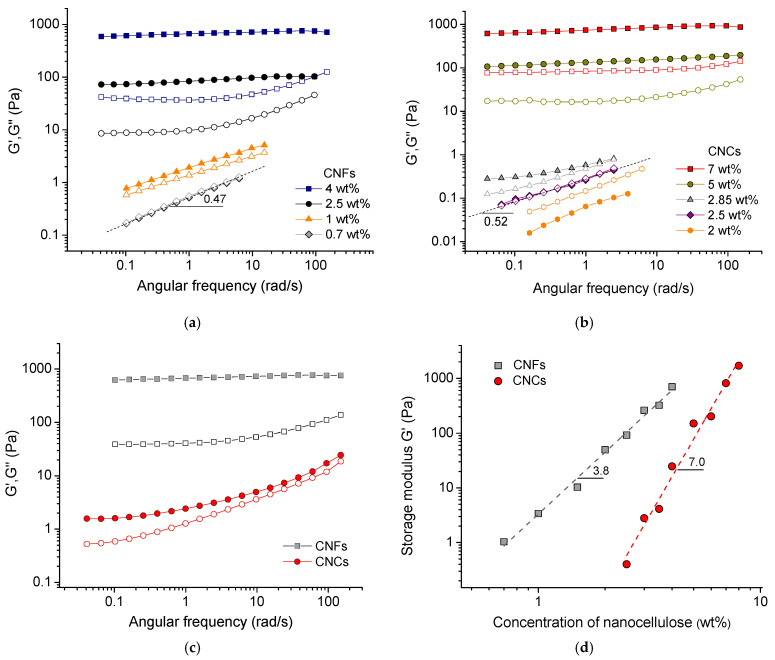
(**a**) Frequency dependencies of storage G′ (filled symbols) and loss G″ (open symbols) moduli for aqueous suspensions of CNFs of different concentrations indicated in Figure. (**b**) Frequency dependencies of storage G′ (filled symbols) and loss G″ (open symbols) moduli for aqueous suspensions of CNCs of different concentrations indicated in Figure. (**c**) Frequency dependencies of storage G′ (filled symbols) and loss G″ (open symbols) moduli for 3.5 wt% aqueous suspensions of CNFs (squares) and CNCs (circles). (**d**) Evolution of storage modulus G′ (at 4.6 rad/s) with CNF (squares) and CNC (circles) concentration.

Figure 4a shows the frequency dependencies of the storage G’ and loss G” moduli for aqueous suspensions at different concentrations of nanofibrils. At a CNF concentration of 0.7 wt%, the G’(*ω*) and G”(*ω*) curves almost coincide with each other, indicating the gel point [50]. The slope of these curves (0.47 ± 0.02) is close to 0.5, which is expected for the gel point [50]: G’(*ω*) = G”(*ω*) = *Kω*^1/2^, where K is a temperature-sensitive factor. Although this approach to determining the gel point was originally developed for chemically crosslinked gels, it has later been shown [50] that it can also be applied to transient networks of colloidal particles. At 1 wt% CNFs, G′ is only slightly larger than G″. At higher CNF concentrations, the G′ value becomes much larger than the G″ value, indicating the dominance of elastic response, and the storage modulus G′ becomes almost independent of frequency, demonstrating a wide plateau typical for network structures. Thus, at these concentrations, the network of percolated CNFs is formed, which is consistent with literature data [4].

As for nanocrystals, at 2 wt% CNCs, the suspension is in a liquid-like state: G” is higher than G’ at all studied frequencies (Figure 4b). A gel point, where G’(*ω*) = G”(*ω*) = *Kω*^1/2^ over a very wide frequency range [50], is observed at 2.5 wt% CNCs (Figure 4b). Therefore, the gel point for CNCs is shifted to a higher concentration compared to CNFs, as expected given their shorter length. To the best of our knowledge, this is the first comparison of the gel point for CNCs and CNFs. It shows that, for CNCs that are at least 10 times shorter than CNFs, the gel point occurs at a concentration that is about 3.5 times higher than for CNFs. At 2.85 wt% CNCs, G’ is larger than G” and highly frequency dependent (Figure 4b), indicating that the network structure in CNC suspension is weak. At 5 wt% CNCs, G’ increases considerably, becoming almost independent of frequency and much higher than G” (Figure 4b), suggesting a strengthening of the gel structure.

Figure 4d shows the variation in storage modulus G′ with nanoparticle concentration for the CNF suspension. One can see that the storage modulus increases by three orders of magnitude with an increase in CNF concentration from 0.3 to 4 wt%. The storage modulus of CNFs follows the scaling dependence G′ ~ C^3.8^, with a power law exponent n of 3.8, which falls within the broad range of reported experimental values: 2.4 [41], 3 [40], 4.5 [51], and 5.2 [52]. It also coincides with the recent theoretical predictions (n = 3.67–7), which attribute the elastic energy of CNFs to bending [53]. For CNCs, the increase in storage modulus with the concentration of nanocrystals is much more significant: G′ ~ C^7.0^ (Figure 4d). This value being in accordance with the previously reported one [26] may be due to the growth of CNC fibrillar-like aggregates occurring at increasing concentration of nanocrystals, which was demonstrated by cryo-EM and cryo-electron tomography [26]. In CNFs, the aggregation is less pronounced than in CNCs because of a steric hindrance effect caused by the kinks in the fibrils [39].

Across the entire range of concentrations studied, the CNF suspensions have a higher storage modulus G’ than the CNC suspensions (Figure 4). For instance, for the 3.5 wt% concentration, the G’ value (at 0.1 rad/s) for CNFs is approximately 280 Pa, while for CNCs, it is only 1.57 Pa, which is a two-order magnitude lower (Figure 4c). A similar difference in G’ values reaching two orders of magnitude was already reported previously [40]. At a 2.5 wt% concentration, the difference is even more significant, exceeding 700 times: G’ (at 0.1 rad/s) is 73.3 Pa for CNFs and 0.096 Pa for CNCs (Figure 4a,b). This can be attributed to the high length of fibrils, which form a stronger entangled network compared to the percolated network of short CNCs, which is not too far from the C* value.

**Figure 5:**
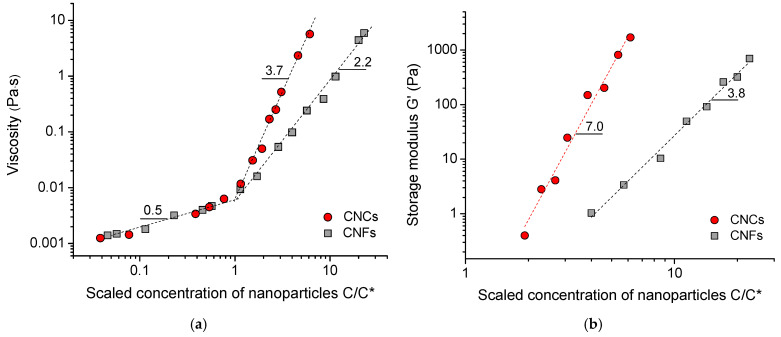
(**a**) Dependence of viscosity of suspensions on CNF and CNC concentration relative to the overlap concentration C* at shear rate of 45 s^−1^. (**b**) Dependence of storage modulus G′ on CNF and CNC concentration relative to C* at angular frequency of 4.6 rad/s.

**Figure 6:**
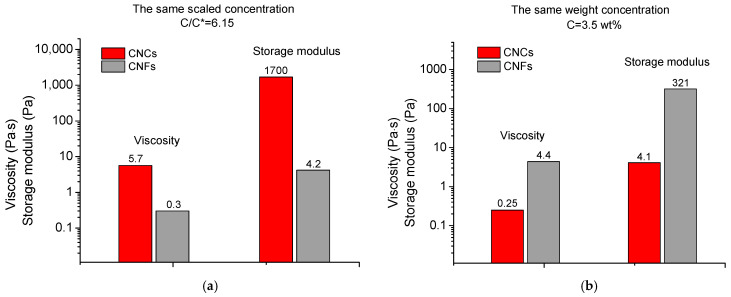
(**a**) Histogram demonstrating the viscosity (at 45 s^−1^) and storage modulus G′ (at 4.6 rad/s) for 1.07 wt% CNF and 8 wt% CNC suspensions having the same C/C* ratio of 6.15. (**b**) Histogram demonstrating the viscosity (at 45 s^−1^) and storage modulus G′ (at 4.6 rad/s) for CNF and CNC suspensions at the same weight concentration of 3.5 wt%.

But if we compare the rheological data for CNCs and CNFs at the same concentration of nanoparticles relative to C*, we obtain an opposite effect: for CNCs, viscosity in the semidilute regime and storage modulus are higher than those for CNFs (Figure 5 and Figure 6). For instance, Figure 6a compares the data at C/C* = 6.15 that corresponds to 8 wt% of CNCs and 1.07 wt% of CNFs. The G′ (at 4.6 rad/s) for CNC suspension is 1.7 kPa, whereas for CNF suspension it is 400-fold lower (G′ = 4.2 Pa). Similarly with viscosity, the viscosity at 45 s^−1^ for the CNF suspension (η = 0.3 Pa·s) is 19-fold lower than for the CNC suspension (η = 5.67 Pa·s). Therefore, the higher viscosity and elasticity observed in CNF suspensions as compared to CNC ones at the same weight concentration of nanoparticles (Figure 6b) are indeed due to a more entangled CNF network. When comparing CNF and CNC suspensions at the same concentration of nanoparticles relative to C*, the situation is reversed (Figure 6a), which may be mainly due to the higher rigidity and intrinsic strength of the highly crystalline CNCs, as well as their ability to aggregate with each other.

### 2.3. After Crosslinking

#### 2.3.1. Phase Behavior

For crosslinking, the concentration of cellulose nanoparticles was fixed at 3.5 wt%. The concentration of multivalent Ca^2+^ ions varied from 5 to 40 mM for CNFs and from 5 to 72 mM for CNCs. At higher concentrations of the crosslinker, syneresis was observed during gel aging. Within 3–7 days after the addition of the crosslinker, the gel slightly shrank and expelled a small amount of water solution. In the case of the crosslinking of macromolecules in a semidilute solution, it has been suggested that syneresis occurs when the number of crosslinks exceeds the initial number of contacts between the macromolecules prior to the addition of the crosslinker [54]. Perhaps a similar explanation applies when, instead of polymer chains, polymer nanocrystals form a gel. The Ca^2+^/COO^−^ molar ratio at the onset of syneresis in 3.5 wt% suspensions was equal to 0.9 for CNFs and 1.8 for CNCs. It implies that, despite the same weight concentration, the initial number of contacts between nanoparticles in a CNF suspension is twice as large. This is expected, given their longer length and some flexibility, which provide more entanglements.

#### 2.3.2. Thermodynamics of Interactions of Cellulose Nanoparticles with Ca^2+^

**Figure 7:**
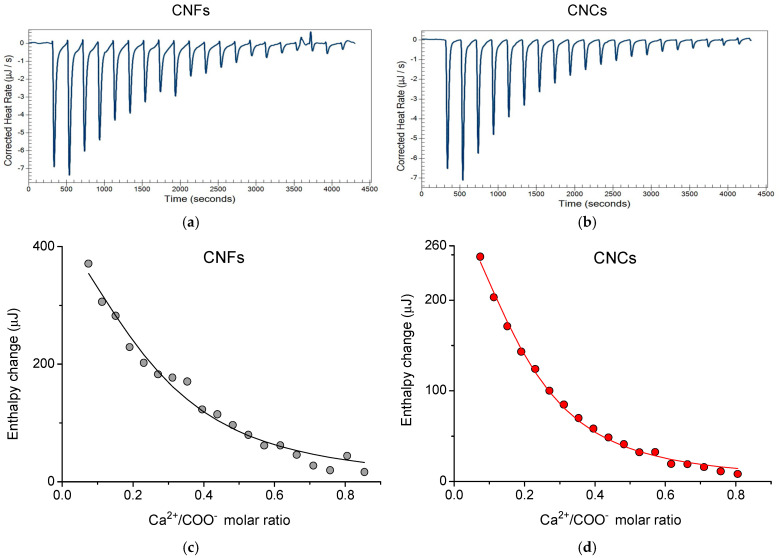
(**a**,**b**) The thermograms of isothermal titration obtained by the subsequent injection of 50 μL of 30 mM calcium chloride to 170 μL of 1 wt% suspensions of CNFs (**a**) and CNCs (**b**) at 293 K. (**c**,**d**) The thermodynamic profiles of isothermal titration of CNFs (**c**) and CNCs (**d**) with Ca^2+^ ions for the same system. The solid lines are fittings of the profiles using an independent binding site interaction model.

The thermodynamics of the interaction of CNFs and CNCs with Ca^2+^ was studied by isothermal titration calorimetry (ITC). This is the first ITC study of the interaction of CNFs with Ca^2+^. Previous research was focused only on the CNCs/Ca^2+^ system [26,54]. The obtained thermograms for CNFs and CNCs (Figure 7a,b) demonstrate the endothermic peaks with each addition of CaCl_2_ solution. In the case of CNCs, these peaks were associated with the adsorption of Ca^2+^ cations onto the negatively charged nanoparticles [55]. According to FTIR spectroscopy data [20,26], both in carboxymethylated CNFs and CNCs, the COO^-^ groups are involved in the interaction with Ca^2+^. Therefore, the area of each peak on the thermogram, corrected by subtracting the heat of dilution, was plotted as a function of the Ca^2+^/COO^−^ molar ratio on the thermodynamic profiles (Figure 7c,d).

From Figure 7a,b, one can see that the thermograms for CNFs and CNCs are quite similar. The fitting of the corresponding thermodynamic profiles (Figure 7c,d) with the independent binding site model also yields similar parameters. They are presented in Table 1. One can see that in both systems, the change in Gibbs free energy ΔG is negative, which indicates that the binding of Ca^2+^ ions onto oppositely charged nanoparticles is spontaneous [56]. The positive values of the enthalpy ΔH and entropy ΔS changes suggest that the binding is entropically driven [57], which is probably due to the release of structured water molecules surrounding the interacting cellulose nanoparticles and Ca^2+^ ions [55]. The release of water molecules is consistent with DFT modeling results of the CNF/Ca^2+^ system [20] that predicts negative values of displaced solvation volume, that is, the decrease in the fibril surface accessible to the solvent upon interaction with the cations.

The estimated values of the stoichiometric numbers *n* for CNFs and CNCs are the same within the experimental error, indicating that 1 mole of Ca^2+^ interacts with 4–5 moles of COO^−^ groups. This observation seems to be reasonable, since CNFs and CNCs contain the same amount of charged carboxylate groups on the surface, as was estimated by conductometry (Figure 1). The ITC results obtained are consistent with the data previously reported for the adsorption of Ca^2+^ on carboxymethylated CNCs [26,55]. In the present study, we show that the same thermodynamic parameters characterize the interaction of Ca^2+^ with carboxymethylated CNFs.

#### 2.3.3. Dynamic Mechanical Properties

**Figure 8:**
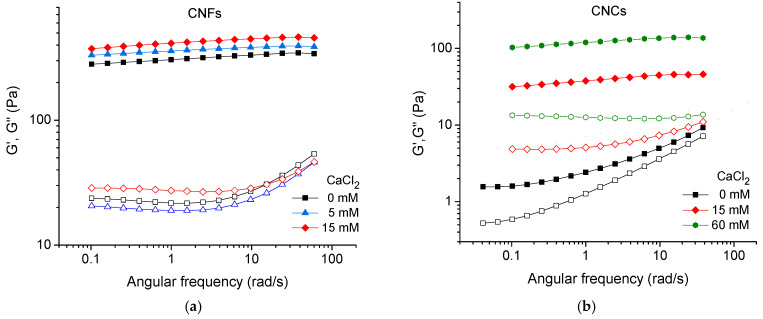

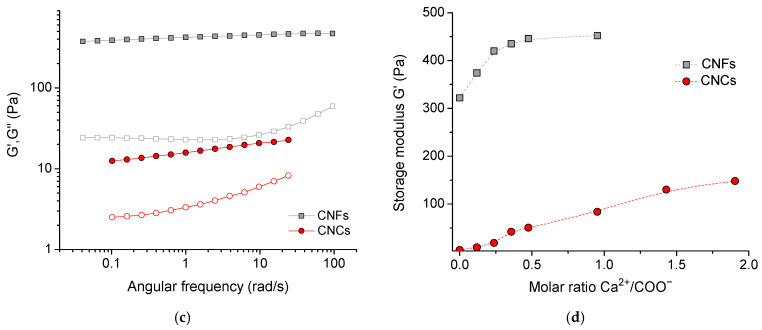
(**a**) Frequency dependencies of storage G′ (filled symbols) and loss G″ (open symbols) moduli for suspensions containing 3.5 wt% CNFs and different concentrations of crosslinker CaCl_2_ indicated in Figure. (**b**) Frequency dependencies of storage G′ (filled symbols) and loss G″ (open symbols) moduli for suspensions containing 3.5 wt% CNCs and different concentrations of crosslinker CaCl_2_ indicated in Figure. (**c**) Frequency dependencies of storage G′ (filled symbols) and loss G″ (open symbols) moduli for suspensions containing 3.5 wt% CNFs (squares) or CNCs (circles) and 10 mM crosslinker CaCl_2_, corresponding to Ca^2+^/COO^−^ molar ratio of ca. 0.25. (**d**) Storage modulus G′ at oscillatory frequency of 4 rad/s as function of Ca^2+^/COO^−^ molar ratio for 3.5 wt% suspensions of CNFs (squares) and CNCs (circles).

In the absence of the crosslinker, the storage modulus G′ of 3.5 wt% CNF suspension is an order of magnitude higher than the loss modulus G” and is almost independent of frequency (Figure 8a). This indicates that the CNFs form a network even without the crosslinker. The addition of the crosslinker leads to an increase in storage modulus G′ by up to 40% (Figure 8a), which suggests the formation of new elastically active elements in the network. These new elastically active elements appear as a result of the crosslinking, which proceeds through electrostatic interactions of negatively charged COO^−^ groups of nanofibrils with divalent Ca^2+^ cations, as was shown previously by DFT calculations [22] and FTIR spectroscopy [20]. Note that the input of new crosslinks to the storage modulus G′ is relatively small, suggesting that at these conditions, the entanglements of CNFs mainly contribute to the elasticity of the CNF network.

As for 3.5 wt% suspension of CNCs, before the addition of multivalent cations, its storage modulus G’ is somewhat higher than G”, and it is strongly dependent on frequency (Figure 8b). This suggests the formation of a weak network. When the crosslinker is added, the storage modulus increases by up to 37 times, becomes an order of magnitude higher than the loss modulus and almost independent of frequency (Figure 8b), indicating a very pronounced strengthening of the network structure. So, the significant impact of crosslinks on the storage modulus suggests that Ca^2+^-crosslinks play a crucial role in the elasticity of the CNC network at this CNC concentration. Most probably, they not only link nanocrystals together but also help incorporate more CNCs into the network structure.

Note that similar behavior (the low values and strong frequency dependence of G’ before the addition of a crosslinker, and the strong increase in G’ and its only slight frequency dependence afterward) was also observed in [20] for CNF suspension at a low concentration of nanofibrils (1.27 wt%). This allows us to assume that the reason for the difference in the behavior of 3.5 wt% suspensions of CNFs and CNCs in the present paper is mainly due to the different C/C* ratios in these suspensions. The CNF suspension, which has a C/C* value of 20, is in a highly entangled state, so that additional crosslinks only have a moderate effect on its elasticity. In contrast, the CNC suspension, with a C/C* value of 2.7, is not too far from C*. Additionally, the CNCs are more rigid than CNFs. As a result, they form only a loose network without a crosslinker, and the effect of the crosslinker on G’ is very significant (similar to the case of 1.27 wt% CNFs with a C/C* value of 7.2).

Although the increase in G’ induced by multivalent ions is much more pronounced for CNCs, when we compare the absolute values of the storage moduli for CNFs and CNCs at the same concentration of crosslinker (Figure 8c,d), we can see that they are always much higher for CNFs, regardless of the amount of crosslinker. Figure 8d shows that the storage modulus of CNF gels increases with the increasing amount of crosslinker, and levels off at a Ca^2+^/COO^−^ molar ratio of ca.0.5. In contrast, the storage modulus of CNC gels continues to increase up to a Ca^2+^/COO^−^ molar ratio of ca. 1.8, a point at which syneresis begins. Probably, in the case of CNCs, the crosslinker not only builds a network of percolated nanocrystals but also links adjacent crystals parallel to each other [25].

#### 2.3.4. Viscosity Recovery

**Figure 9:**
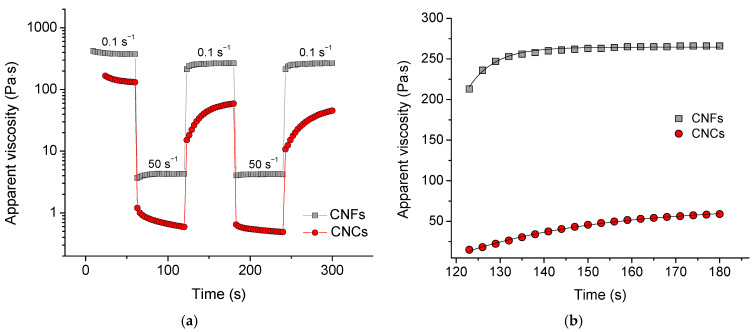
(**a**) Viscosity recovery after periodic variation in shear rate (50 s^−1^ for 60 s, 0.1 s^−1^ for 60 s, etc.) for suspensions containing 3.5 wt% CNFs (gray) or CNCs (red) and 15 mM CaCl_2_; (**b**) Fitting of viscosity recovery with exponential function for second cycle of periodic variation in shear rate for suspensions containing 3.5 wt% CNFs (gray) or CNCs (red) and 15 mM CaCl_2_.

Self-assembled nanocellulose networks, formed by non-covalent interactions, are expected to be reversible. To demonstrate this, the viscosity recovery was investigated. The experiments involved applying alternating high (50 s^−1^) and low (0.1 s^−1^) shear rates (for 60 s each) over several cycles [58]. When the shear rate was increased to 50 s^−1^, the viscosity decreased by 2 orders of magnitude because the network was disrupted and the nanoparticles aligned along the direction of flow. When the shear rate was reduced to 0.1 s^−1^, the viscosity recovered, as the network was reformed (Figure 9a).

The recovery curves (Figure 9b) can be fitted with an exponential function, allowing us to determine the recovery time τ [59]:

$$\eta =a+b\exp\left(-\frac{t}{\tau }\right)$$

Here, t is time, a, b are coefficients. It was found (Table 2) that the recovery time for the CNF network is only slightly affected by the amount of crosslinker, while for CNCs, it significantly decreases with increasing crosslinker concentration. This finding is consistent with our suggestion that the entanglements of CNFs are primarily responsible for the formation of the CNF network and thus determine the recovery time. At the same time, the contribution of Ca^2+^-crosslinks to the elasticity of the CNC network is very considerable. For this reason, an increased amount of the crosslinker helps to fix the recovered structure more quickly.

The most important finding is that the recovery time for the CNF network is up to nine times shorter than that of the CNC network, implying that the entanglements are restored faster than the Ca^2+^-crosslinks. For the CNF network, it only takes about 6 s to rebuild its structure after a disruption. Table 2 shows that for both CNFs and CNCs, the recovery time in the third cycle is longer than in the second cycle. This means that it is more difficult to rebuild the network structure, which has only recently recovered.

The recovery is not complete, as the viscosity of the reconstructed network is slightly lower in each subsequent cycle (Figure 9a). Table 2 shows that the percent recovery for CNFs depends only slightly on the concentration of crosslinker, whereas for CNCs, it significantly increases with increasing crosslinker concentration. This is also consistent with our hypothesis that the entanglements of CNFs mainly contribute to the formation of the CNF network, and therefore, they determine the percentage of recovery. In contrast, the contribution of the Ca^2+^-crosslinks to the elasticity of the CNC network is very pronounced, as they help to incorporate more CNCs into the network structure. For this reason, a higher number of crosslinks leads to a more complete network reconstruction. The maximum percent recovery (83–85%) is almost the same for CNFs and CNCs, although CNCs require a much higher concentration of crosslinker and a longer time (Table 2). Therefore, CNFs are more effective than CNCs in situations where a fast recovery of rheological characteristics is required.

## 3. Conclusions

In the present paper, it was shown that CNFs have some differences compared to CNCs: (i) they have high viscosity and storage modulus at low concentrations, (ii) they form gels (without crosslinker) at lower concentrations. These differences are primarily due to their longer length providing a lower C* concentration. However, at the same C/C* ratio, CNCs acquire a much higher viscosity and storage modulus than CNFs, which is mainly due to their higher crystallinity-induced rigidity and intrinsic strength, as well as their ability to aggregate with each other.

Adding divalent cations strengthens the networks of both CNFs and CNCs, but the CNF networks remain stronger in the presence of a crosslinker as well. The Ca^2+^-crosslinked networks are shown to be reversible. Although a maximum percent recovery (83–85%) is almost the same for CNFs and CNCs, CFCs require a much smaller concentration of a crosslinker and a shorter time to achieve it.

Since both CNC and CNF networks can easily be destroyed by shear and then quickly reform, this opens up new possibilities for their use in a variety of applications where this property is highly desirable, such as 3D extrusion printing and the production of injectable hydrogels. In 3D extrusion printing, the ink must have its viscosity reduced under shear in order to easily flow through the nozzle of the printhead. However, once it has been deposited at its intended location, it must quickly regain its original high viscosity in order to maintain the desired shape without spreading or deformation [16]. In biomedicine, this property is required for injectable hydrogels [60]. When these hydrogels are destroyed by shear, they can be injected into the body in liquid form, causing minimal damage to surrounding tissues. Once inside the body, they regain their structural integrity and promote tissue regeneration by providing mechanical support and allowing for the spatiotemporal delivery of cells or therapeutic agents [60].

The provided comparison of CNFs and CNCs can help to make a proper choice of nanocellulose material for a particular application. The wider use of these materials would benefit from the development of cost-effective and scalable production methods through exploring alternative raw materials and treatment processes.

## 4. Materials and Methods

### 4.1. Materials

Carboxymethylated CNFs (product CNF-CM-P) and carboxymethylated CNCs (product CNC-CM-SD) from Cellulose Lab (Fredericton, NB, Canada) were used without further purification. CNFs were provided in the form of a 4 wt% aqueous gel. According to the information given by the supplier, the degree of crystallinity of CNFs is less than 30%, the length L of nanofibrils is 1–3 microns, and their average diameter D is about 4 nm.

CNCs were provided as a spray-dried powder with a density of 1.5 g/cm^3^, a degree of crystallinity of 86–90%, and a zeta potential of −40 mV. In our previous study [26], it was determined using transmission electron microscopy that individual CNCs have an average length L of 90 nm and an average diameter D of 5 nm.

Calcium chloride (purity > 97%) from Sigma-Aldrich (St. Louis, MO, USA) was used as received. Water was purified with a Millipore Milli-Q system (Millipore, Milford, MA, USA).

### 4.2. CNF Suspensions and Hydrogel Preparation

To obtain a CNF suspension of the required concentration, water was added to a 4% solution of CNFs and stirred on a magnetic stirrer. Then a solution of crosslinker CaCl_2_ was added and stirred on a magnetic stirrer for 1 h at 250 rpm. The prepared samples were left for 1 day to equilibrate.

### 4.3. CNC Suspensions and Hydrogel Preparation

To obtain a CNC suspension, water was added to a CNC powder and ultrasonic treatment (5 min, 100 W) was performed with a Sonics VCX-500 Vibra-Cell ultrasonic homogenizer (Cole Palmer, Vernon Hills, IL, USA). Then the solution of crosslinker CaCl_2_ was added and stirred on a magnetic stirrer for 1 h at 650 rpm. The prepared samples were left for 1 day to equilibrate.

### 4.4. Rheometry

The rheological measurements were carried out on a Physica MCR 301 rheometer (Anton Paar, Graz, Austria) at 20 °C. Two cells were used: a cell with double-gap cylindrical geometry (diameter of 26.4 mm, height of 40 mm, gap of 0.42 mm), and a cell with cone–plate geometry (diameter of 40 mm, cone angle of 2°). The samples were loaded into the cells for 10 min. prior to the experiment to allow for equilibration. The experiments were conducted at a deformation of 1%, which corresponds to the linear viscoelastic regime, as identified by amplitude sweep tests at 1 rad/s [61].

### 4.5. Isothermal Titration Calorimetry

The ITC measurements were performed using a Nano ITC isothermal calorimeter (TA Instruments, New Castle, DE, USA) at 293 K. The sample cell was loaded with 1 wt% CNC or CNF suspensions. For the titration of the suspensions with Ca^2+^ ions, a 30 mM aqueous solution of CaCl_2_ was loaded into a syringe. In total, 20 injections of titrant (2.5 μL each) were performed into 170 μL of CNC or CNF suspensions with a 20 min time lag at a mixing rate of 200 rpm. The raw data of the CNC or CNF titration with cations were corrected by subtracting the corresponding thermograms of cation titration into the distilled water (Figure S2). The peaks in the corrected thermograms were integrated to receive corresponding thermodynamic profiles representing enthalpy change vs. molar ratio of cations to COO^−^ groups of nanocellulose. The first peak in each thermogram was ignored. The thermodynamic parameters of binding interaction were determined by fitting the profiles with an independent binding site interaction model, which implies the binding of ions to similar binding sites of the nanocellulose [62]. Practically, the fitting of the profiles with the Wiseman isotherm [63] was performed using the NanoAnalyze 3.12.0 software (TA Instruments, New Castle, DE, USA).

### 4.6. Conductometric Titration

The conductometric titration was performed on an Aquasearcher AB23EC stationary conductometer (Ohaus, Moscow, Russia) as follows [35]. First, 494.3 mL of a dilute suspension containing 0.3 g of CNFs was prepared using deionized water. Next, 0.7 mL of 1 M HCl and 5 mL of 0.1 M NaCl were added to exchange the sodium counterions with hydrogen ions and adjust the conductivity to a measurable range. In the case of CNCs, 10 mL of suspension containing 0.1 g of CNCs was sonicated for 10 min, and 3 mL of 0.1 M HCl and 5 mL of 0.1 M NaCl were added to the mixture. Then the deionized water was added to adjust the total volume of the suspension to 200 mL.

The CNF and CNC suspensions prepared in this way were titrated with a 0.1 M NaOH solution. The content of carboxylic groups was determined from the volume of NaOH consumed between the strong acid and weak acid equivalence points (Figure 1). The equivalence points were identified by finding the intersection of the linear fits of the acidic and basic regions of the titration curve with the linear fit of the wide plateau between them.

### 4.7. Potentiometric Titration

The potentiometric titration of CNC suspension was performed on a Seven Multi Mettler Toledo (Greifensee, Switzerland) pH meter as follows [35]. First, 10 mL of suspension containing 0.1 g of CNCs was sonicated for 10 min, and then 187 mL of deionized water was added. Next, 3 mL of 0.1 M HCl was added to the mixture to set the pH to 3. The total volume of the suspension was equal to 200 mL. Under constant stirring, the CNC suspension was then titrated with 0.01 mL additions of 0.1 M NaOH using the pH meter. To determine the equivalence point, the point on the titration curve with the steepest slope corresponding to the largest value of the first derivative was determined (Figure S3, Supporting Information). To calculate the pK_a_ from the titration curve, the volume of NaOH added at the equivalence point, V, was divided by 2 to obtain the half-equivalence point. The pH under these conditions is considered as a pK_a_ value [36].

### 4.8. Polarized Optical Microscopy

Polarized optical microscopy studies were conducted using a Nikon Eclipse LV100 POL polarized light microscope (Nikon, Tokyo, Japan), equipped with a CFI TU Plan Fluor Epi objective lens (p 5x), at room temperature. The suspensions were poured between two glass plates for measurements.

## Figures and Tables

**Table 1:** Enthalpy ΔH and entropy ΔS changes, stoichiometric number *n*, binding constants K_d,_ and Gibbs free energy change ΔG obtained from thermodynamic profiles of CNFs and CNCs titration with Ca^2+^ ions at 293 K upon fitting with independent binding sites model.

Sample	ΔH, kJ/mol	*n*	K_a_, mmol^−1^	ΔS, J/mol·K	ΔG, kJ/mol
CNFs	9 ± 5	0.24 ± 0.08	0.57 ± 0.30	83 ± 3	−15 ± 1
CNCs	6 ± 1	0.18 ± 0.03	0.83 ± 0.25	77 ± 3	−16 ± 1

**Table 2:** Recovery time and percent recovery at periodic variation in shear rate for suspensions containing 3.5 wt% CNFs and CNCs and different concentrations of CaCl_2_.

Concentration of CaCl_2_, mM	Recovery Time τ, s		Percent Recovery	
	Second Cycle	Third Cycle	Second Cycle	Third Cycle
CNFs				
5	5.8	7.7	83	82
10	5.7	7.1	72	70
15	6.0	6.6	72	72
20	6.3	7.2	80	83
CNCs				
10	-	-	33	23
15	31.2	58.8	45	35
20	18.9	31.1	48	41
40	12.1	12.5	55	52
60	11.8	15.9	76	72
80	8.4	9.3	83	85

## Data Availability

The original contributions presented in this study are included in the article/Supplementary Materials. Further inquiries can be directed to the corresponding author.

## Supplementary Materials

The following supporting information can be downloaded at: https://www.mdpi.com/article/10.3390/gels11110926/s1, Figure S1: Polarized optical microscopy images of CNF suspensions with different concentrations of nanofibrils indicated in Figure; Figure S2: The thermograms obtained at isothermal titration of Ca^2+^ ions into water at 293 K; Figure S3: First derivative of the titration curve of 0.05 wt% aqueous suspension of CNCs (0.1 g, in acid form) with 0.1 M NaOH to determine the equivalence point. The volume of added 0.1 M NaOH corresponding to equivalence point is 2.97 mL.
