# Supplementary material for: Cellulose Nanofibrils vs Nanocrystals: Rheology of Suspensions and Hydrogels
Source: Gels. 2025 Nov 19;11(11):926. doi: 10.3390/gels11110926 (PMC12652647; doi:10.3390/gels11110926)
Supplement: Supplementary file 1 [file gels-11-00926-s001.zip › gels-3991138-supplementary.pdf]

gels

MDPI

Supplementary materials

for

Cellulose Nanofibrils vs. Nanocrystals: Rheology of Suspensions and Hydrogels

Alexander S. Ospennikov, Alexander L. Kwiatkowski and Olga E. Philippova

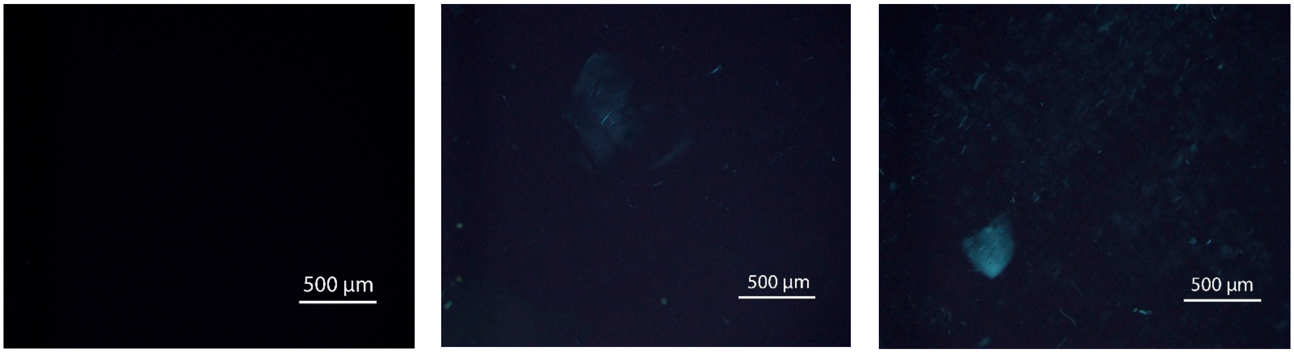

1.3 wt% (**a**) 1.4 wt% (**b**) 2.5 wt% (**c**)

Figure S1. Polarized optical microscopy images of CNF suspensions with different concentrations of nanofibrils indicated in Figure.

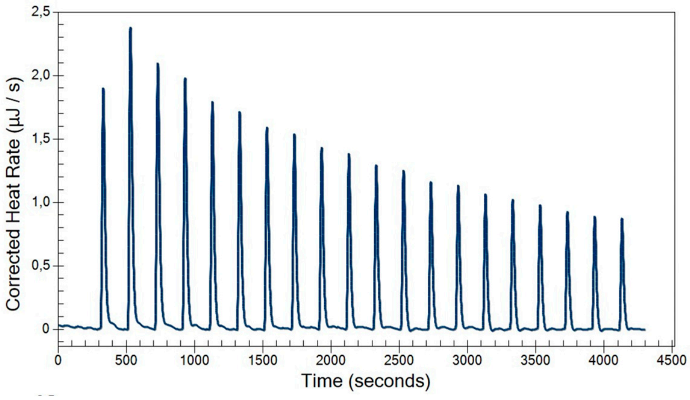

Figure S2. The thermograms obtained at isothermal titration of Ca^{2+} ions into water at 293 K.

 https://doi.org/10.3390/gels11110926

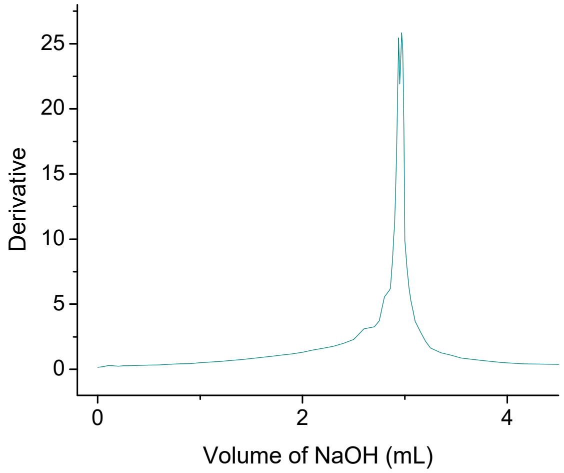

Figure S3. First derivative of the titration curve of 0.05 wt% aqueous suspension of CNCs (0.1 g, in acid form) with 0.1 M NaOH to determine the equivalence point. The volume of added 0.1 M NaOH corresponding to equivalence point is 2.97 mL.
